# Poxvirus Antigen Staining of Immune Cells as a Biomarker to Predict Disease Outcome in Monkeypox and Cowpox Virus Infection in Non-Human Primates

**DOI:** 10.1371/journal.pone.0060533

**Published:** 2013-04-05

**Authors:** Haifeng Song, Krisztina Janosko, Reed F. Johnson, Jing Qin, Nicole Josleyn, Catherine Jett, Russell Byrum, Marisa St. Claire, Julie Dyall, Joseph E. Blaney, Gerald Jennings, Peter B. Jahrling

**Affiliations:** 1 Integrated Research Facility, National Institute of Allergy and Infectious Diseases, National Institutes of Health, Frederick, Maryland, United States of America; 2 Emerging Viral Pathogens Section, National Institute of Allergy and Infectious Diseases, National Institutes of Health, Bethesda, Maryland, United States of America; 3 Biostatistics Research Branch, National Institute of Allergy and Infectious Diseases, National Institutes of Health, Bethesda, Maryland, United States of America; Emory University School of Medicine, United States of America

## Abstract

Infection of non-human primates (NHPs) such as rhesus and cynomolgus macaques with monkeypox virus (MPXV) or cowpox virus (CPXV) serve as models to study poxvirus pathogenesis and to evaluate vaccines and anti-orthopox therapeutics. Intravenous inoculation of macaques with high dose of MPXV (>1–2×10^7^ PFU) or CPXV (>10^2^ PFU) results in 80% to 100% mortality and 66 to 100% mortality respectively. Here we report that NHPs with positive detection of poxvirus antigens in immune cells by flow cytometric staining, especially in monocytes and granulocytes succumbed to virus infection and that early positive pox staining is a strong predictor for lethality. Samples from four independent studies were analyzed. Eighteen NHPs from three different experiments were inoculated with two different MPXV strains at lethal doses. Ten NHPs displayed positive pox-staining and all 10 NHPs reached moribund endpoint. In contrast, none of the three NHPs that survived anticipated lethal virus dose showed apparent virus staining in the monocytes and granulocytes. In addition, three NHPs that were challenged with a lethal dose of MPXV and received cidofovir treatment were pox-antigen negative and all three NHPs survived. Furthermore, data from a CPXV study also demonstrated that 6/9 NHPs were pox-antigen staining positive and all 6 NHPs reached euthanasia endpoint, while the three survivors were pox-antigen staining negative. Thus, we conclude that monitoring pox-antigen staining in immune cells can be used as a biomarker to predict the prognosis of virus infection. Future studies should focus on the mechanisms and implications of the pox-infection of immune cells and the correlation between pox-antigen detection in immune cells and disease progression in human poxviral infection.

## Introduction

MPXV and CPXV are members of the *Orthopoxvirus* family that also includes variola virus (VARV), the causative agent of human smallpox and vaccinia virus (VACV). After cessation of broad VACV immunization, MPXV infections are now emerging as a public health concern. MPXV induces smallpox-like symptoms and has up to a 10% case fatality rate, especially in the younger unvaccinated population [1], [2]. In addition, VARV or MPXV could also be released as bioterrorist agents [3]. Such concerns have resulted in increasing effort to elucidate poxvirus pathogenesis and to develop anti-orthopox virus vaccines and drugs. Many animal models have been developed for such research purposes [4]. Among them, non-human primates (NHPs) infected with MPXV or CPXV serve as models of human orthopox viral disease [5]–[8].

Human MPXV infection can cause severe illness, even lethality [2], [6], [9]. Therefore, an early biomarker to predict the outcome of the infection will be critical in determining effective treatment in human clinics and in studies with animal models. Historically, monitoring disease progression largely depends on clinical scoring systems such as the sum of major clinical manifestations including fever intensity and lesion number etc. [10], [11]. However, clinical scoring is not always a reliable indicator of clinical progression and does not correlate with dose of inoculated virus and disease severity [12], [13]. Thus, a biological marker for infection prognosis is needed. Using an ectromelia virus (a murine poxvirus) challenge model in mice, several biomarkers such as the serum concentration of alanine aminotransferase (ALT), interferon-gamma (IFN-γ), and copies of poxvirus DNA in the mouse blood were used to monitor disease progression and antiviral efficacy of the hexadecycloxypropyl ester of cidofovir (CMX001) [14]. Administration of CMX001 was associated with a reduced serum concentration of alanine ALT, IFN-γ, and genome copies of ectromelia virus compared with those observed in untreated mice. However, elevations in serum concentrations of ALT in untreated MPXV-infected NHPS have not been consistently observed [12], [15], [16] and therefore the usefulness of ALT as a marker of antiviral efficacy is questionable.

Although some biological markers such as blood genomic DNA copies of virus may prove to be helpful in monitoring antiviral efficacy of therapeutic interventions [15], no early prognostic indicators have been found to predict outcome in untreated MPXV-infected NHPs. A previous *in vitro* study with fresh human peripheral blood leukocytes (PBLs) demonstrated that specific populations of PBLs were differentially infected with GFP-labeled vaccinia virus. The monocyte population was most frequently infected, followed by B cells, NK cells and T cells [17]. *In vivo* studies combined with immunohistochemistry analysis also reported that phagocytes/monocytes are the cells that most likely carry poxviruses and were speculated to help the virus spread in blood to other tissues [5]. However, no effort has been made to clearly characterize the immune cells that are infected with MPXV in animal models with specific cell markers. In the current studies, we monitored the presence of poxvirus or viral antigens in immune cells by an intracellular staining method in NHPs infected with lethal doses of MPXV or CPXV. We found blood monocytes and granulocytes were the major cells to be positive for pox-antigen staining with flow cytometry. The positive detection of poxvirus antigens in immune cells closely correlates with disease progression. Furthermore, early detection of pox-positive immune cells was predictive of NHPs reaching moribund endpoint in MPXV-infected or CPXV-infected NHPs.

Our findings provide a useful tool to monitor clinical progression and the prognosis of MPXV or CPXV infection and to evaluate the efficacy of drugs and vaccines.

## Materials and Methods

### Animals, Virus Inoculation and Sample Collection

MPXV Zaire 79 and Sierra Leone virus stocks were propagated in BSC-1 cells at a multiplicity of infection (MOI) of 0.1 for 3 days. Virus inoculum was prepared by three freeze/thawing cycles followed by centrifugation and ultrasonic treatment, then pelleted over a 36% sucrose cushion. CPXV Brighton strain was propagated in Vero E6 cells at a multiplicity of infection (MOI) of 0.1 for 3 days. Virus was pelleted over 36% sucrose cushion.

Rhesus macaques of both sexes, ranging from 4–8 kg, were housed in bio-containment cages (Primate Products, Miami, FL) and were acclimated to a 12∶12 hour light/dark cycle in a temperature and humidity controlled, AAALAC, International accredited vivarium facility at NIH. Nonhuman primates were maintained in accordance with National Institute of Allergy and Infectious Diseases (NIAID) SOPs, and had water and food (Teklad Monkey Diet, Harlan Laboratories, Indianapolis, IN) provided *ad libitum*. Animals were provided environmental enrichment in accordance with National Institute of Health (NIH) enrichment SOPs. All animals were acclimated to the study facility for a minimum of two weeks prior to the start of the study. Prior to enrollment, NHPs were screened and found to be seronegative for simian retrovirus, simian T cell leukemia, VACV, CPXV, and MPXV. Inoculations were performed by intravenous injection of desired virus doses in 1 ml PBS.

The data for MPXV infections reported here are results from NHPs that were infected intravenously in three individual experiments (Table 1). Experiment A included four NHPs infected with 1.5×10^7^ PFU of Sierra Leone. In experiment B, our analysis included the eight NHPs in two different groups that received lethal doses of MPXV. Five NHPs were infected with 1.5×10^7^ PFU Zaire 79 strain of the Central Africa clade and three NHPs were infected with 2.5×10^8^ PFU of Sierra Leone from the West Africa clade. Six NHPs were from experiment C with three infected with 5×10^7^ Zaire 79 and another three were inoculated with Zaire 79 and also received the anti-pox drug, cidofovir, at days −1, 1, 3 5, 7, 10, and 13 pre- or post-inoculation at 5 mg/kg body weight, a dose that has been shown to protect NHPs from death caused by challenge with lethal dose of MPXV [18]. In addition to NHPs inoculated with MPXV, we also included data from a Cowpox virus study in Cynomolgus monkeys. Three groups of three NHPs were intravenously inoculated with CPXV at 5×10^2^ PFU, 5×10^3^ PFU, and 5×10^4^ PFU.

**Table 1:** NHP groups in the study.

Experiment	Virus	Inoculation route	Inoculationdose (PFU)	Cidofovir	No. of NHPs	Moribund	Survivors
A	MPCXV Sierra Leone	iv	1.5×10^7^	no	4	4	0
B	MPXV Sierra Leone	iv	2.5×10^8^	no	3	2	1
	MPXV Zaire79	iv	1.5×10^7^	no	5	4	1
C	MPXV Zaire 79	iv	5×10^7^	no	3	2	1
	MPXV Zaire 79	iv	5×10^7^	yes	3	0	3
D	CPXV	iv	5×10^2^	no	3	2	1
	CPXV	iv	5×10^3^	no	3	2	1
	CPXV	iv	5×10^4^	no	3	2	1

Research was strictly conducted in compliance with the *Guide for the Care of Laboratory Animals*, the Animal Welfare Act regulations, and all applicable NIH Policies. All animal experiments were approved by NIAID, Division of Intramural Research, Animal Care and Use Committee. All animal handling and scientific procedures were performed under anesthesia by injecting 10–25 mg/kg IM Ketamine hydrochloride. Investigators and animal care personnel provided additional comfort and care such as supplemental heat, highly palatable food items such as fruit, baby carrots, peanuts, Ensure®, Gatorade® and IV, SQ, or oral fluids, consistent with the scientific integrity of the protocol. Animals that were not eating were tube-fed under sedation. Animals which were tube-fed were given metoclopramide 0.3 mg/kg IM 15 minutes prior to tube feeding to prevent vomiting. Morbidity sacrifice endpoints were used to determine euthanasia time points. Animals were euthanized in accordance with the 2007 AVMA Guidelines on euthanasia utilizing exsanguination via direct cardiac puncture following induction of deep anesthesia by IV injection of Sodium pentobarbital (100 mg/kg). Death was ensured by administration of sodium pentobarbital (Fatal Plus®, Vortech Pharmaceuticals, Dearborn, MI) IV and followed by a full necropsy.

### Reagent and Antibodies

The following antibodies and reagents were purchased from BD Biosciences (San Jose, CA): CD3-Pacific Blue, AlexaFluor 700 (clone SP34-2); CD8-FITC, -APC, -Pacific Blue (clone SK1); CD16-PE, -APC (clone 3G8); CD14-Pacific Blue (clone M5E2); CD20-APC, -PE-Cy7 (clone L27); and CD4-PE (clone L200). NKG2A-PE (clone Z199) was purchased from Beckman Coulter (Miami, FL). Fc Block was from Miltenyi Biotec (Auburn, CA). Goat anti-Rabbit IgG AlexaFluor-750 and Yellow LIVE/DEAD® dye were from Invitrogen (Carlsbad, CA); Rabbit anti-vaccinia (pox) and rabbit IgG were purchased from Accurate Chemical & Scientific Corporation (Westbury, NY).

### Preparation of Leukocytes or PBMCs

To obtain leukocytes, 0.2 ml whole blood was suspended in 1 ml ACK buffer (Invitrogen) for 2–3 minutes followed by centrifugation and two washes with PBS containing 2% fetal bovine serum (FBS). To prepare PBMCs, EDTA-blood tubes were centrifuged at 1000×g for 10 min to get plasma. The remaining part was diluted in 2-3-fold PBS+2% FBS and layered over 100% Ficoll. PBMCs were centrifuged at 1000×g room temperature for 30 min. The interface mononuclear cell layer was collected and washed twice with PBS+2% FBS. The leukocytes or PBMCs were either directly used for FACS staining or frozen in −80°C for future study.

### Surface Staining and Intracellular Staining

0.5–1×10^6^ PBMCs or leukocytes were incubated with a mixture of antibodies to cell surface makers for 15–20 min at room temperature. After washing twice with FACS buffer, the cells were fixed with Cytofix/Cytoperm (BD Biosciences) for 30–40 minutes at room temperature. The cells were washed with cytoperm/cytowash (BD Biosciences), followed by blocking with Fc receptor antibody and by staining with rabbit anti-vaccinia Ab, either at room temperature for one hour or leave at 4°C for overnight. The cells were washed and the secondary antibody goat-anti-rabbit IgG-AlexaFluor 750 was applied and incubated at RT for 1 hr. After washing with Cytoperm/cytowash, the cells were suspended in cytoperm/wash buffer and acquired using LSR-Fortessa cytometer (Becton Dickinson, San Diego, CA). The data was analyzed with Flowjo 9.3.1software (TreeStar Inc. Ashland, OR) or with BD FACS Diva software. The staining result is expressed as frequency of positive staining in the cell population of interest. To obtain the staining frequency of pox-antigen of a specific sample, the value of the isotype control staining of the same sample was subtracted from the value of pox-antigen specific staining. We define a frequency >0.2% as the threshold for positive staining as isotype control staining is approximately ≤0.2. When a staining gave a frequency of around 0.2%, we examined the original FACS plot to confirm the staining intensity of the apparently positive population.

### Quantification of Viral Load in the Whole Blood by qPCR

Viral load in whole blood was determined by quantitative PCR (qPCR) after DNA isolation from 50 µl of whole blood using Genfind v2 according to the manufacturer’s directions (Agencourt, Danvers, MA). Isolated DNA was eluted in 120 uL of 1XTE, diluted 1/5, and screened for the presence of the poxvirus HA gene with a LightCycler apparatus (Roche, Basel, Switzerland). 5 µl of diluted DNA was incubated with 15 µl of reaction mix (10 µl of MasterMix, 4.5 µl of ultrapure water, and 0.5 µl primers directed at the HA [B2R] gene) [19]. Thermal cycling for the LightCycler was performed as follows: 95°C for 2 minutes, followed by 40 cycles of 95°C for 1 second and 60°C for 20 seconds. A synthetic oligo was made by Integrated DNA Technologies (Coralville, IA) and used as the assay standard. Results were calculated by reference to the standard curve, multiplied by the appropriate dilution factor, and reported as gene copies per ml. The lower limit of detection of the synthetic oligo was 10 copies, while the upper limit was 1×10^8^ copies per ml. We set our limit of detection at 10,000 gene copies/ml so that we were well within the standard deviations that would account for dilution errors and extraction efficiencies.

### Statistical Analysis

Due to the small sample size in each experiment in MPXV study, the statistical analysis was performed in two approaches. First, a 2 by 2 table (moribund or survivor vs. pox-staining positive or negative) for each individual experiment was generated (Table 2), followed by Cochran-Mantel-Haenszel test. In this analysis, a cut-off of 0.2% of staining frequency was used as the staining positive threshold. Secondly, a stratified Cox regression model was used to analyze the raw data of staining frequency from all three MPXV studies together. For CPXV study, Fisher exact test was used in statistics analysis for the pox-staining result. A Cox proportional hazard model was used to analyze the qPCR data from both MPXV and CPXV studies.

**Table 2:** Statistics 2 by 2 table (moribund or survivor vs. pox-staining positive or negative).

Experimental groups	poxvirus	NHP fate	*Pox-staining positive (>0.2%)at day 2 (MPXV) or day 9 (CPXV)	Pox-staining negative (≤0.2%) atday 2 (MPXV) or day 9 (CPXV)
A	MPXV	moribund	4	0
		survivor	0	0
B	MPXV	moribund	4	2
		survivor	0	2
C	MPXV	moribund	2	0
		survivor	0	4
D	CPXV	moribund	6	0
		survivor	0	3

* The frequency of pox-staining is expressed as the subtraction value of pox-specific staining frequency in granulocyte and monocytes gate by isotype control value. Positive staining is defined as >0.2%.

## Results

### Intracellular Staining Identifies Poxvirus Antigen Positive Cells

**Figure 1:**
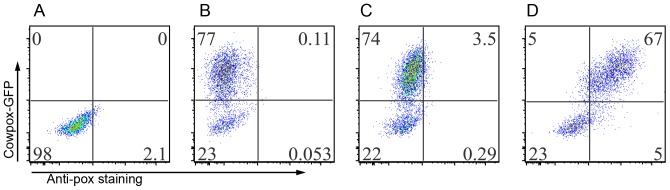
Detection of poxvirus proteins by FACS intracellular staining. Hela-S3 cells were infected with GFP-CPXV overnight. The cells were harvested and fixed with BD cytofix/perm, followed by intracellular staining. A), Hela-S3 cells without infection, no staining; B), cells with GFP-CPXV infection, but no staining; C), cells with GFP-CPXV infection with isotype control antibody staining; and D), cells with GFP-CPXV infection with anti-vaccinia antibody staining. Data were acquired with BD LSR-Fortessa and analyzed with software Flowjo 9.3.1 (Tree-Star, Ashland, Oregon).

Models of MPXV infection in rhesus or cynomolgus macaques have been used to evaluate anti-poxvirus drugs and vaccines because disease observed in macaques resembles that seen in humans. One of the important questions of poxvirus pathogenesis is whether immune cells and which immune cell populations can be infected. To answer this question, we developed an assay for intracellular staining of poxvirus infection with a polyclonal anti-vaccinia antibody. We infected Hela-S3 cells with GFP-labeled cowpox virus [20] and stained with anti-vaccinia antibody. As shown in figure 1, all GFP-positive cells were also positive by staining with anti-vaccinia Ab, indicating that the anti-vaccinia antibody specifically stain for pox-virus (Figure 1D). In contrast, the infected cells stained with isotype Ab control (Figure 1C) or without staining (Figure 1B) showed GFP single positive, further confirming the sensitivity and specificity of the anti-vaccinia Ab. Thus, the staining method is reliable for identifying cells either with active viral replication or that have acquired poxvirus antigen.

### Monocytes and Granulocytes/Neutrophils are the Major Immune Cell Populations that Stained Positive for Poxvirus Antigen

**Figure 2:**
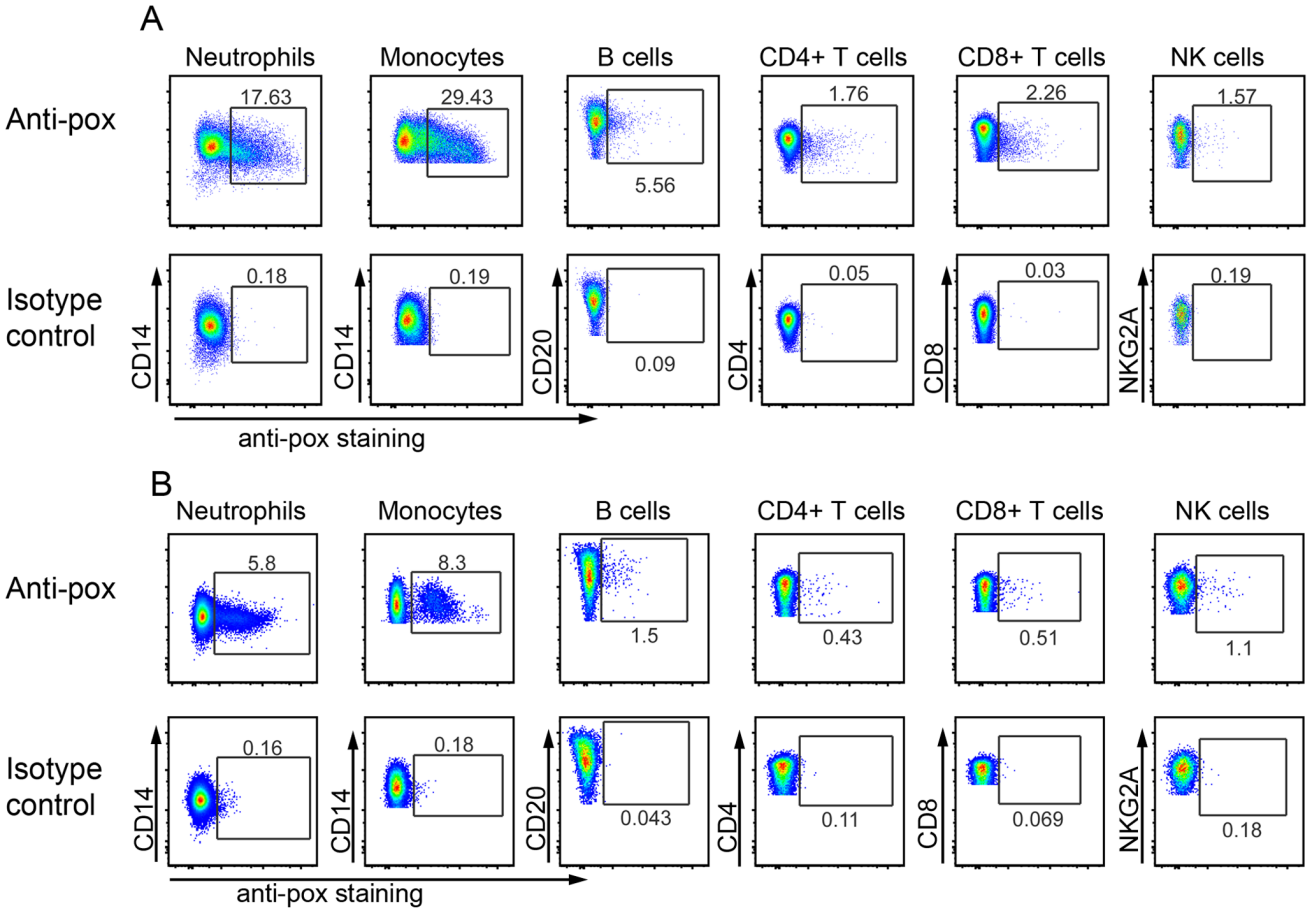
Granulocytes/neutrophils and monocytes are the major cell populations stained positively for poxvirus proteins. A), representative staining of pox-antigen staining from MPXV-infected NHPs. Leukocytes from day 4 infected NHP were prepared and stained with cell markers and anti-pox antibody as described in materials and Methods. The figure showed pox-specific staining (upper row) and isotype antibody control (lower row) staining of different cell populations. The granulocytes/neutrophils were gated based on FSC/SSC distribution. Neutrophil staining is displayed as CD14+ (Y-axis) vs. pox+ (x-axis) population. From the FSC/SSC plot, the lymphocytes and monocytes were gated as one population. Then, CD3-CD14+ monocytes, CD3+CD4+ T cells, CD3+CD8+ T cells, CD3-CD20+ B cells were further defined within the “lymphocytes+monocytes” population. NKG2A+ NK cell analysis was gated on CD3-CD8+ lymphocytes. B), representative staining of NHP infected with CPXV at day 9 post virus inoculation from experiment D. The gating strategy is the same as in Figure 2A.

**Figure 3:**
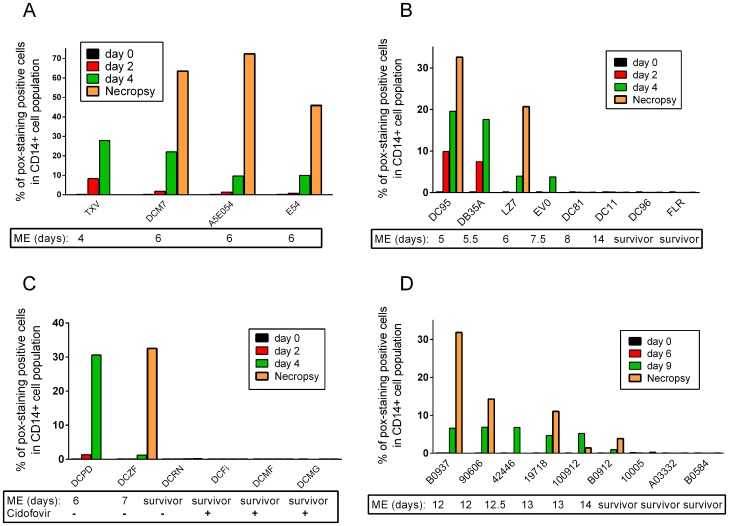
NHPs with positive pox-staining succumbed to MPXV or CPXV infection. Leukocytes from individual NHPs at different time points were stained for cell surface markers and pox-antigen. The frequency of positive pox-staining cells in granulocytes/monocytes gating is displayed in the Figures. Figure 3A, B, and C shows results from the three MPXV experiments, and D, displays data from CPXV study. ME: Moribund Endpoint.

We next sought to determine which immune cell populations are positive for poxvirus by staining. Leukocytes from NHPs infected with various lethal doses of MPXV were stained with cell surface markers combined with intracellular anti-pox staining. Our result showed that among all the cell populations, monocytes and granulocytes/neutrophils are the predominant cells exhibiting positive pox-antigen staining (Figure 2A). However, the frequency of pox-positive monocytes and granulocytes varied (Figure 3, Table 3). For example, in experiment A, the peak frequency of pox-positive granulocyte and monocytes reached as high as 72.4% at the time of necropsy (NHP A5E054). However, the peak frequency observed in experiment B is 32.55% at necropsy (DC95). In addition to granulocytes and monocytes, lymphocyte populations such as CD20+ B cells, NK cells, CD4+ T cells, and CD8+ T cells also stained positive for pox-antigen (>0.2%) compared to isotype control albeit at very low frequency (Figure 2, Table 3). The frequency of pox-antigen positive CD20+ B cells and NK cells tended to be low at day 2 and day 4, but mounted to as high as around 10% at necropsy days in some moribund NHPs. However, the frequency of infected CD4+ T cells and CD8+ T cells remained low throughout the study (Table 3).

**Table 3:** Frequency of positive pox-antigen immune cells in the blood.

Experiment	DatePost- inoculation	Mean frequency of pox-antigen positive cells in different cell populations (range)				
		Granulocyte & Monocytes*	CD4 cells	CD8 cells	CD20 cells	NK cells
A	Day 2	3.11 (0.78–8.35)	0.14 (0.04–0.38)	0.22 (0.04–0.62)	1.55 (0.61–2.3)	0.11 (0–0.21)
	Day 4	17.4 (9.6–27.9)	0.60 (0.13–1.7)	0.97 (0.27–2.2)	3.83 (1.3–5.4)	0.62 (0.2–1.4)
	day 6	60.6 (45.9–72.4)	1.44 (1.39–1.47)	1.16 (0.97–1.42)	7.78 (5.6–9.37)	8.77 (5.8–12.1)
B	Day 2	4.56 (0.3–9.91)	0.14 (0–0.54)	0	0.46 (0.05–1.18)	0.90 (0–2.53)
	Day 4	11.23 (3.79–19.58)	0.24 (0–0.41)	0.38 (0–0.81)	1.14 (0–2.63)	2.13 (0.1–4.39)
	day 8 or Necropsy**	26.62 (20.68, 32.55)	0.31 (0, 0.62)	0.05 (0,0.18)	1.11 (0.67, 1.54)	7.75 (5.5, 9.7)
C	Day 2	0.72 (0.1–1.33)	0.03 (0.03, 0.03)	0.03 (0.03, 0.03)	0.72 (0.1, 1.33)	0.16 (0.16, 0.16)
	Day 4	15.9 (1.2–30.6)	0.39 (0.1, 0.68)	0.77 (0.14, 1.39)	1.97 (0.1, 3.84)	2.07 (0.9, 3.24)
	Day 8 or Necropsy**	32.5	0.36	1.1	2.7	4.75
D	Day 9	5.16 (0.92–6.84)	0.03 (0–0.15)	0.11 (0–0.25)	0.56 (0–1.1)	0.40 (0.07––1.12)
	Day 12	10.78 (1.42–31.85)	0.14 (0–0.22)	0.11 (0–0.21)	1.3 (0.12–2.1)	1.13 (1.0–1.22)

* granulocytes and monocytes cannot be clearly distinguished by FSC/SSC gating in some samples when virus infection is severe. Thus the two populations were gated together as one population for analysis. Value is the subtraction of isotype control staining from the pox-antigen staining result. The data does not include result from survivors, which all gave a value <0.2%.

** for the NHPs that reached moribund after day 8, day 8 analysis was included in the table. Otherwise, the data from necropsies between day 5 and day 8 was included.

We also examined the immune cells for pox-staining from NHPs that were infected with CPXV. The result demonstrated a similar pattern to that from MPXV studies (Figure 2B, Table 3). We found that granulocytes and monocytes are also the two major cell populations to stain positive for poxvirus antigens (Figure 2B, Table 3).

### All NHPs with Positive Pox-staining in their Monocytes and Granulocytes Reached Moribund Endpoint

In the experiments with MPXV, more than 80% of NHPs inoculated with a lethal dose of MPXV succumbed to infection between days 4 and 14 post-inoculation. We studied the possible correlation between the disease progression/moribund endpoint and the pox-antigen staining of immune cells.

Since monocytes and granulocytes/neutrophils are the major cell populations that stained positive for pox antigen in the blood and the frequency of pox-positive T cells, B cells, and NK cells in the blood were low (Figure 2, Table 3), we focused on the monocytes and neutrophils in the analysis of circulating immune cells.

18 NHPs that received a lethal dose of MPXV from 3 different experiments were included in the evaluation (Table 1). We determined the time of the first appearance of pox-antigen positive immune cells following virus inoculation. Immune cells became pox-antigen positive in MPXV-NHPs as early as day 2 following virus inoculation and the frequency of pox-positive cells gradually increased until NHPs reached moribund endpoint (Figure 3 and table 3). For example, for NHP DC95 in experiment B, the frequency of pox-positive, CD14+ cells (including monocytes and granulocytes) at day 2, day 4 and day 5 were 9.91%, 19.58% and 32.55% respectively (Figure 3B). In contrast, for NHPs intravenously infected with low dose of CPXV, pox-positive immune cells were first detected at day 9 after virus inoculation (Figure 3D).

Poxvirus staining from MPXV-infected NHPs showed that the immune cells especially monocytes and neutrophils from all 4 NHPs in experiment A were positive from day 2 to day 6 and all four NHPs reached moribund endpoint. Four NHPs in experiment B, regardless of the virus strain they received, were pox-antigen positive in their granulocytes and monocytes from day 2 to day 7 post inoculation and all four NHPs also succumbed to virus infection. The two survivors, one from Zaire 79 group (DC96) and one from Sierra Leone group (FLR) in experiment B, did not demonstrate clear poxvirus staining in their granulocytes and monocytes at any time points. In experiment C, two NHPs were pox-antigen positive and both reached moribund endpoint. The survivor (DCRN) from experiment C and the three NHPs that received MPXV and cidofovir were pox-antigen negative throughout the experiment. It is important to point out that two NHPs in experiment B (DC11 and DC81) also reached moribund endpoint at day 8 and day 14 respectively and were pox-antigen negative throughout the study (Figure 3B), suggesting that other factors may also contribute to disease severity and outcome. Thus, ten of twelve subjects that reached moribund endpoint from three experiments stained positive for pox antigens in their monocytes and granulocytes. In contrast, the 6 survivors including the 3 cidofovir treated NHPs were pox-antigen negative. The data suggests that the virus antigen detection in monocytes and granulocytes may predict disease progression and outcome.

We then analyzed the data from a CPXV study. Among the 9 NHPs inoculated with three different doses of CPXV (5×10^2^, 5×10^5^, 5×10^4^PFU), six NHPs independent of virus doses were pox-antigen positive at day 9 and all six NHPs reached moribund endpoint between day 12 and 14. In contrast, the three survivors were either transiently pox-antigen positive (NHP#10005 at day 12, 0.22%) or were negative (Figure 3D). Together, the data leads to the conclusion that the detection of pox virus antigen in monocytes and granulocytes correlates with disease progression and moribund endpoint.

### Early Detection of Pox-antigen Positive Immune Cells Correlates with Disease Progression and Early NHPs Reaching Moribund Endpoint

During our analysis on samples from MPXV-infected NHPs, we noticed that MPXV-positive monocytes and granulocytes can be detected as early as day 2 post infection and the frequency of MPXV-positive cells gradually increased until moribund endpoint. Those NHPs with early detection of pox-antigen positive immune cells and with higher frequency of pox-antigen positive cells at early time points tended to have more severe disease and reached moribund endpoint more rapidly. For example in experiment B, NHPs DC95 and DB35A had detectable pox-antigen positive cells (granulocytes and monocytes ) at day 2 with a frequency of 9.91% and 7.44% and the frequency of positive staining cells increased at day 4 (19.58 and 17.61% respectively). These two NHPs succumbed to infection at day 5 and day 5.5. In contrast, pox-staining positive cells first clearly appeared at day 4 in NHP LZ7 and EV0 (3.95% and 3.79%) and the NHPs reached moribund at day 6 and day 7.5 (Figure 3B). In experiment A (Figure 3A), the frequency of pox-antigen positive cells at day 2 were 8.4%, 1.9%, 1.4%, and 0.8% for NHPs TXV, DCM7, A5E054,and E54 respectively and reached to 32.5%, 22.1%, 9.6%, and 10% at day 4. TXV reached moribund at day 4 and the other three at day 6. In experiment C, NHP DCPD was first detected as MPXV-staining positive in immune cells at day 2 and NHP DCZF at day 4. DCPD reached moribund at day 6 and DCZF at day 7 (Figure 3C). The data from CPXV study showed similar pattern. The NHPs with relatively higher frequency of pox-positive staining cells at day 9 such as NHP B0937, 90606, and 42446 reached moribund at day 12, while the one with a lower infected cell frequency at day 9 (B0912) reached moribund at day 14 (Figure 3D).

We performed statistical analysis to see if early detection of positive pox-staining correlates with disease progression and early NHP moribund endpoint. Cochran-Mantel-Haenszel test was performed based on the 2 by 2 table (Table 2) with a hypothesis that there is no association between positive pox-staining (we define 0.2% as the cut-off for positive staining based on isotype control background) at day 2 and moribund. However, the p value was 0.014 (p = 0.014), which rejects the null hypothesis, indicating a correlation between early detection of positive pox-staining and animals reaching a moribund state. Secondly, the stratified Cox regression analysis with all the raw data from MPXV studies gave an estimated hazard ratio of 4.19 (95% CI is 1.34 to 51.19, p<0.001), indicating every one unit (%) of increasing of pox-positive staining cells on day 2 would lead to 4.19 times possibility of NHP reaching moribund criteria.

Fisher exact test used in the statistics analysis for CPXV study showed that day 9 pox-staining result correlates with animals reaching a moribund state (p = 0.0119).

Taken together, the data indicate that positive pox-staining results at an early detection date (day 2 for MPXV and day 9 for CPXV) predicts more severe disease progression and an increased likelihood that the NHP met moribund endpoint criteria. Statistical analysis on day 4 result from MPXV studies also showed a correlation between pox-antigen positive staining results and disease progression (p = 0.013). From the data observed in our experiments, once the NHP shows positive staining for poxvirus compared to isotype control (>0.2%), it will succumb quickly within a period of 2–6 days following the first time of detection of positive pox-staining cells. Result from the CPXV study demonstrated similar pattern. The first day to detect positive pox-antigen staining cells in the current CPXV study was day 9. All the NHPs with positive pox-antigen staining of immune cells succumbed between day12 and 14.

### Comparison of Pox Antigen Staining of Immune Cells by Flow Cytometry and Detection of Blood Viral DNA Copies Measured by qPCR as Potential Biomarkers

In orthopox virus studies, blood viral load is measured by qPCR and is used as an indicator for disease severity [15],[21],[22], however, direct correlation has not been established. We therefore studied the possible correlation between blood viral load and moribund endpoint and were interested in the comparison between pox-staining method and qPCR in the context of NHP moribund endpoint. We observed that most NHPs with positive pox-antigen staining in their immune cells at day 2 or day 4 also had early positive detection of pox genomic DNAs in the blood (Table 4 and 5). However, a few samples tested positive for FACS staining of immune cells on day 2, but below detection limit by qPCR and vice versa (Table 4 and 5). For example, for survivors FLR, DC96, and DCRN, pox-staining of immune cells gave negative results for all time points. However, they were positive by qPCR at day 2 or later time points. Similar results were observed for NHPs DCFi, DCMF, and DCMG from experiment C. While FACS staining did not give a clear positive for those samples at early or later time points, qPCR was negative in early time points day 2 and day 4, but became positive at later time point on day 7 to day 16 (Experiment C, NHPs DCFi, DCMF, and DCMG, data not shown). Statistics analysis indicated that day 2 qPCR result is not a predictor of survival (p = 0.19). In contrast, the day 4 qPCR result correlated with disease progression (p = 0.01). A similar correlation between virus load and disease progression was observed from the CPXV study with a hazard ratio of 3.66 (95% confidence interval 1.03, 13.07). The data indicates that virus load measured by qPCR may be predictive to disease progression, but it could be difficult to determine at which time point data will be predictive. In contrast, as soon as pox-antigen is detected by FACS staining, it is predictive of disease progression. Therefore, we conclude that FACS staining of poxvirus antigen in immune cells is a better predictor for disease progression.

**Table 4:** NHP outcome, pox-antigen staining of immune cells and blood poxvirus DNA copy number at early time points.

Experiments	Virus/PFU	NHP ID	Date of euthanasia	Poxvirus staining by FACS*		Poxvirus DNA copy number by qPCR, log10 gc/ml	
				Day 2	Day 4	Day 2	Day 4
A	SL1.5×10^7^	TXV	4	8.35	27.9	5.03	7.37
		DCM7	6	1.88	22.1	OOR***	5.72
		A5E054	6	1.41	9.63	4.17	6.12
		E54	6	0.78	10	4.26	6.03
B	SL2.5×10^8^	DC95	5	9.91	19.58	5.24	7.4
		DBA35	5.5	7.44	17.61	5.4	7.1
		FLR	survival	0.05	0.1	4.4	5.1
B	Zaire 79	LZ7	6	0.1	3.95	4.4	5.6
	1.5×10^7^	EV0	7.5	0.1	3.79	4.4	5.9
		DC81	8	0.12	0.1	OOR	4.4
		DC11	14	0.15	0.05	OOR	4.4
		DC96	survival	0.05	0.08	OOR	4.4
C	Zaire 79	DCPD	6	1.33	30.2	OOR	7.21
	8.5×10^7^	DCZF	7	0.1	1.2	OOR	5.93
		DCRN	survival	0.1	0.15	OOR	5.51
C	Zaire 79	DCFi**	survival	0.09	0	OOR	OOR
	8.5×10^7^	DCMF**	survival	0.09	0	OOR	OOR
	+ Cidofovir	DCMG**	survival	0.02	0	OOR	OOR

* , % of positive pox-staining cells in granulocyte and monocyte gate. The number is the subtraction of isotype control staining from the pox-antigen staining result. A value below 0 is expressed as 0.

** , these three NHPs showed positive qPCR result at later time points from day 7 to day 16. The data is not shown in the table.

*** , OOR, out of range low.

**Table 5:** Comparison of FACS pox-staining and plasma PCR for pox-DNA number in CPXV study.

NHP ID	Date of euthanasia	Poxvirus staining by FACS(% of granulocytes+monocytes)*		Poxvirus DNA copy number by qPCR,log10 gc/ml	
		Day 6	Day 9	Day 6	Day 9
B0937	12	0	6.5	OOR**	5.8
90606	12	0	6.84	OOR	6.2
42446	12.5	0	6.78	4.0	7.2
19718	13	0	4.68	OOR	5.8
100912	13	0	5.21	3.4	6.6
B0912	14	0	0.92	4.0	5.5
10005	survivor	0	0	OOR	4.7
A0332	survivor	0	0	OOR	5.1
B0584	survivor	0	0.07	4.6	5.5

* , % of positive pox-staining cells in granulocyte and monocyte gate. The number is the subtraction of isotype control staining from the pox-antigen staining result. A value below 0 is expressed as 0.

** , OOR, out of range low.

## Discussion

In the current *in vivo* NHP studies, we report for the first time that monocytes and granulocytes in the blood are the major cell populations that were pox-antigen positive measured by FACS. In contrast, only a small proportion of lymphocytes (B cells, NK cells, and T cells) were pox-antigen positive. Most importantly, we have shown that pox-antigen positive granulocytes and monocytes appeared at day 2 to day 7 post MPXV inoculation by iv route and all NHPs with positive pox-staining of immune cells reached moribund endpoint. In addition, statistical analysis showed a positive correlation between pox-antigen positive cells at early time points (day 2 and day 4) and disease progression and NHPs reaching moribund endpoint. Analysis of data from a cowpox virus study in cynomolgus monkeys resulted in similar conclusion. In the CPXV study, pox-antigen positive granulocytes and monocytes appeared at later time points (day 9) compared to MPXV (day 2), probably because of the low dose of virus inoculation. Nevertheless, once the pox-antigen positive cells were detected and remained positive, the NHPs developed more severe symptoms and reached moribund endpoints. In this particular CPXV study, day 9 pox-staining results positively correlated with NHPs reaching moribund endpoints. The results in the CPXV study with lower viral dose may have interesting implication for human cases. Often, human subjects may be infected with low dose of virus and it is difficult to assess the prognosis of an infection. However, if the technique we report here applies to human infection, one may monitor the pox-antigen staining in immune cells. Once a positive staining is observed, it may predict a more severe disease progression and outcome.

In studies of viral pathogenesis and therapeutic development, it is critical to have one or more biomarkers to predict disease progression and infection prognosis. It has been reported that in Ectromelia virus infected mice, the concentration of blood ALT, IFNγ, and viral genomic DNA copy number could be used to monitor disease progression and anti-viral efficacy [14]. However, we did not observe a correlation between plasma IFNγ and ALT level and disease progression in our previous study (unpublished observation) in NHPs. Our current analysis demonstrated that viral load at day 4 post-inoculation correlated with disease progression. However, using qPCR results as an indicator for disease progression has its own limitations. All NHPs included in the current analysis regardless of outcome, were positive with qPCR at certain stages of the experiment post-inoculation. In the MPXV study, viral load at day 2 did not correlate with outcome whereas day 4 viral load did. In a planned experiment, the qPCR data that are of predictive value may be obtained in retrospective analysis. However, during an outbreak, qPCR value would likely be less useful since it will be difficult to determine the time point that may give predictive qPCR data. In addition, the sensitivity of a qPCR assay is largely affected by the starting volume of the blood and the DNA extraction efficiency and in some case it may not be able to detect low quantities of viral genome. Our observation that detection of pox-antigen positive cells in circulating immune cells correlates with disease progression and NHPs reaching moribund endpoint, may provide a simple system to monitor disease progression in NHP orthopox virus models. The intracellular staining procedure can be finished in a few hours and requires only a small amount of blood (100–200 ul). The detection of pox-antigen positive immune cells as early at day 2 after infection will allow us to identify NHPs that will present a more severe disease early and it will help to monitor the disease progressions during an animal study when combined with monitoring the standard clinical symptoms. Most importantly, it provides a sensitive approach to evaluate anti-viral therapeutics and vaccines. If a drug or vaccine can even partially inhibit virus replication and reduce the viral load, it may protect the infected individual from death. This effect can be monitored by a negative staining of poxvirus in immune cells. An example to support this application is the results from the group of NHPs that received both lethal Zaire 79 and cidofovir. All the NHPs in this group showed negative staining of pox-antigen in immune cells and all NHPs survived in contrast to their counterparts that received the same dose of virus but were untreated. Two of three NHPs showed virus staining positive and reached moribund endpoint. Future work needs to be done to determine if this system can be used to predict poxvirus disease progression in humans. Our system may offer a tool to predict the prognosis for human infection in an endemic outbreak of MPXV infection or infection with other orthopox viruses.

Numerous factors may play a role in determining if a cell type can be infected by a virus, such as the genomic expression profile of the cells; the type and number of cell surface receptors; the cell internalization of virus induced by phagocytosis/endocytosis and the permissibility of virus replication within the cells. Our result that monocytes and granulocytes demonstrated higher pox-antigen positive staining than other lymphocyte populations suggested that the successful infection of MPXV and CPXV of immune cells may correlate with their capacity of phagocytosis and endocytosis. However, we cannot rule out the possibility that monocytes and granulocytes express higher level of MPXV-specific receptors that mediate virus entry.

Further study is required to determine if the granulocytes and monocytes from the survivors and the two NHPs that succumbed, but with negative pox-antigen staining in their immune cells had phagocytosis/endocytosis dysfunction; or if virus failed to undergo productive replication. Although our analysis concluded that detection of plasma poxvirus DNA copies is less predictive of disease progression and animal death, it appears that all the NHPs that showed positive pox-antigen staining of immune cells had either an earlier detection or higher copy number of poxvirus genomic DNA than either the NHPs that survived or the two NHPs that died with negative staining of immune cells. It is unknown if the potentially early high level of virus in the blood indicated by genomic DNA copy caused immune cell infection or the immune cell infection resulted in compromised ability of immune system to inhibit/reduce blood virus titer. It is also likely that the high poxvirus DNA copy number in the blood was derived from the poxviruses in the immune cells. In NHPs DC11 and DC81 as well as the three surviving NHPs, FLR, DC96, and DCRN, their blood virus load became higher at later time points (day 8 and day 11). However, the immune cells were pox-antigen negative, suggesting virus load alone is not the solely determinant for immune cell pox-antigen staining result. The timing of the virus titer is especially critical. It is possible that the cell status is also critical in determining its susceptibility to MPXV or CPXV. At day 8 and day 11, although higher virus load exists in the blood, they will not be able to enter the cells most likely because the cells may have altered their cell surface expression profile and become non-permissible to MPXV or CPXV entry or replication. Therefore, the initial few days is critical for the prognosis of the infection.

We also speculate on the cause-and-effect relationship of our findings to the moribund state of the animal. Monocytes and neutrophils are the most important arms in early innate immunity to cope with virus infection. If granulocytes and monocytes are productively infected or have alterations in function due to infection, these two cell populations may alter their ability to kill virus-infected cells. Therefore, virus infection and replication will be unchecked, which could be reflected by the higher copy number of poxvirus DNA. On the other hand, virus infection of monocytes and neutrophils could alter their cytokine secretion profile and the cytokines may facilitate tissue inflammation and polarize immune responses in a harmful direction.
